# Hybrid intelligent strategy driving high-dimensional encrypted orbital angular momentum comb multicasting towards optical data-transmission networks

**DOI:** 10.1038/s41377-026-02386-3

**Published:** 2026-08-05

**Authors:** Shiyun Zhou, Lang Li, Jinyu Yang, Shurui Zhang, Zhiyuan Zhou, Chunqing Gao, Shiyao Fu

**Affiliations:** 1 School of Optics and Photonics, Beijing Institute of Technology, Beijing, 100081 China; 2 Key Laboratory of Photoelectronic Imaging Technology and System, Ministry of Education of the People’s Republic of China, Beijing, 100081 China; 3 Key Laboratory of Information Photonics Technology, Ministry of Industry and Information Technology of the People’s Republic of China, Beijing, 100081 China; 4 Key Laboratory of Quantum Information, University of Science and Technology of China, Hefei, Anhui 230026 China; 5 Synergetic Innovation Center of Quantum Information & Quantum Physics, University of Science and Technology of China, Hefei, Anhui 230026 China; 6 National Key Laboratory on Near-surface Detection, Beijing, 100072 China

**Keywords:** Lasers, LEDs and light sources, Optical techniques

## Abstract

Achieving higher degrees and dimensions of light is one of the accessible paths for increasing the capacity of optical data-transmission systems. Orbital angular momentum (OAM), owing to its infinite and mutually orthogonal nature, offers a promising carrier for high-capacity and high-dimensional data transmission. However, current OAM-based shift keying and multicasting schemes predominantly rely on single-mode encoding or simple mode complexing, which fails to fully exploit the high-dimensional potential of OAM. This limitation primarily arises from the challenge of directly modulating complex multiplexed OAM states—such as OAM combs—which typically requires bulky optical setups and complicated iteration algorithms. Here, a hybrid intelligent strategy for high-dimensional OAM comb multicasting is demonstrated, enabling simultaneous multi-channel transmission with high-dimensional OAM encoding using a single phase-only hologram. By jointly AI-driven OAM comb generation and physics-guided wave-vector manipulation, a phase-only modulation framework is developed to directly tailor multiple structured OAM combs in a single modulation step, significantly improving the photon efficiency of OAM shift keying. The scalability and fidelity of the proposed approach are experimentally validated through 4, 6, and 8-channel multicasting demonstrations. Furthermore, a mixed encoding protocol is introduced to enhance transmission security in one-to-many multicasting scenarios, enabling parallel delivery of distinct image contents to different users. A six-channel OAM comb communication system achieves real-time transmission with a bit-error rate below 7 × 10⁻⁵. Our proposal offers a compact and scalable solution for high-dimensional OAM-based data transmission and provides a promising pathway toward next-generation large-capacity optical networks.

## Abstract (web summary)

We propose a hybrid intelligent strategy driven framework for high-dimensional OAM comb multicasting, enabling simultaneous multi-channel broadcasting with secure hybrid encoding, and significantly enhancing the capacity and efficiency of optical data transmission.

## Graphical abstract

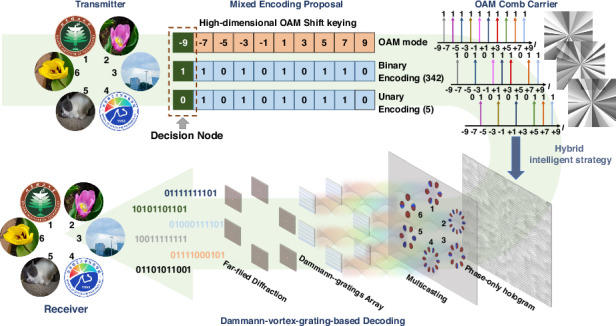

## Introduction

With the exponential growth of global data traffic, modern optical communication systems are facing severe bandwidth limitations^1^. Efficient utilization of light’s degrees and dimensions of freedom is essential for scaling up optical communication capacity^2–4^. High-dimensional encoding schemes have emerged as a promising pathway to enhance spectral efficiency and system scalability^5^. However, traditional multiplexing techniques, such as wavelength-, time-, and polarization-division multiplexing, encounter physical and technical limitations when scaling to higher capacities without adding significant system complexity.

Among various physical dimensions of light, orbital angular momentum (OAM) stands out due to its theoretically infinite and mutually orthogonal eigenstates^6–9^. This property makes OAM an ideal candidate for realizing high-capacity and high-dimensional communication systems^10,11^. Over the past decade, OAM-based data transmission has been successfully demonstrated in both free-space and fibers^12–14^, encompassing OAM shift keying (OAM-SK)^15–19^ and mode-division multiplexing (OAM-DM)^20,21^, showing its strong potential for next-generation optical communication networks. Nevertheless, both OAM-SK and OAM-DM require combining multiple OAM channels into a single transmission link, demanding bulky optical setups, leading to increased hardware complexity, limited scalability, and reduced real-time performance. Moreover, these schemes primarily operate on single-mode or low-order multiplexed OAM states, failing to fully exploit the high-dimensional potential of OAM combs^22–25^, which are structured multi-mode OAM superpositions that can carry significantly richer information.

Unlike common OAM-SK or OAM-DM systems that treat each OAM mode as an independent information carrier, high-dimensional OAM comb multicasting employs structured OAM combs as composite symbols, enabling one-to-many multicasting within a single holographic modulation step. Direct modulation of multiple structured OAM combs, however, remains fundamentally challenging due to their complex spatial distributions^26^ and the need for high-precision dynamic modulation.

To overcome these challenges, in this paper, we introduce a hybrid intelligent strategy^27^ for high-dimensional OAM comb multicasting, implemented through a single-shot holographic modulation framework. By integrating deep learning-based phase optimization with iterative physics-guided refinement, we design phase-only holograms that can simultaneously tailor multiple OAM combs and encode orthogonal OAM modes within a single modulation process. This hybrid strategy effectively mitigates the exponential growth of the phase optimization space as the OAM dimensionality and multicast order increase. Based on this framework, we achieve 1024-ary high-dimensional shift keying across multiple parallel channels, reaching a balance between spectral efficiency and hardware simplicity. Furthermore, we extend a mixed encrypted encoding scheme that jointly integrates binary and unary (single-state) encoding within a unified OAM comb architecture. This mixed encoding strategy not only improves encoding flexibility but also enhances transmission security in one-to-many multicasting scenarios, as decoding cannot be accomplished using common OAM-SK protocols. Experimentally, we demonstrate a six-channel, ten-mode OAM comb communication system, where each channel supports 1024-ary OAM-mode under the mixed encoding scheme. Real-time transmission is achieved with a bit-error-rate (BER) below 7 × 10⁻⁵, approaching error-free performance. Moreover, the mixed encoding strategy exhibits transmission security which is verified through experiment. Our proposal significantly reduces system complexity, enhances modulation scalability, and provides a compact, adaptive, and high-fidelity OAM tailoring mechanism, and represents a scalable and efficient pathway toward high-dimensional OAM communication, with promising implications for large-scale optical networks and advanced spatial-mode photonics.

## Results

### Concept overview

**Fig. 1:**
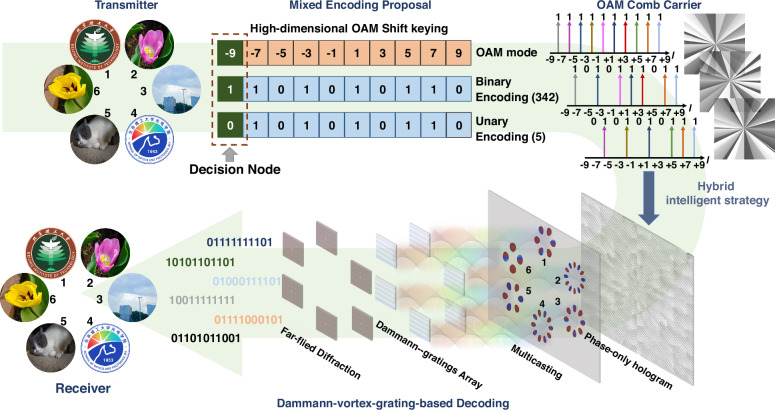
Schematic illustration of the hybrid intelligent OAM comb multicasting system, including mixed encoding proposal, single-shot phase-only OAM comb carrier holographic modulation, and DVG-based demultiplexing for one-to-many high-dimensional data multicasting

Figure 1 illustrates the overall concept and workflow of the proposed hybrid intelligent strategy enabling high-dimensional OAM comb-based encrypted multicasting and decoding. On the transmitter side, multiple messages are assigned to different multicast channels. These messages are first mapped to a mixed-encoding proposal, where a ten-mode OAM comb {−9, − 7, − 5, − 3, − 1, + 1, + 3, + 5, + 7, + 9} acts as the high-dimensional carrier. The encoded message bit patterns are then transformed into phase-only holograms, generated by the proposed hybrid intelligent algorithm. These holograms generate structured OAM comb carriers in a single modulation step, producing multiple OAM comb beams corresponding to different multicast users. On the receiver side, a Dammann-vortex-grating (DVG)-based mode demultiplexing module separates the simultaneously transmitted OAM combs into spatially distinct diffraction orders^28^. Each order corresponds to an independent communication channel. Far-field intensity patterns are recorded, and the encoded symbols are recovered via OAM-spectrum extraction and hybrid decoding. This enables scalable one-to-many parallel OAM comb transmission, supporting multi-channel image delivery, image segmentation transmission, and high-dimensional symbol decoding with low bit-error rates. Overall, the mixed encoding proposal, single-shot phase-only OAM comb holography, and DVG demultiplexing are jointly integrated to create a compact, scalable, and high-dimensional OAM multicasting optical data-transmission system.

### Mixed encoding proposal

**Fig. 2:**
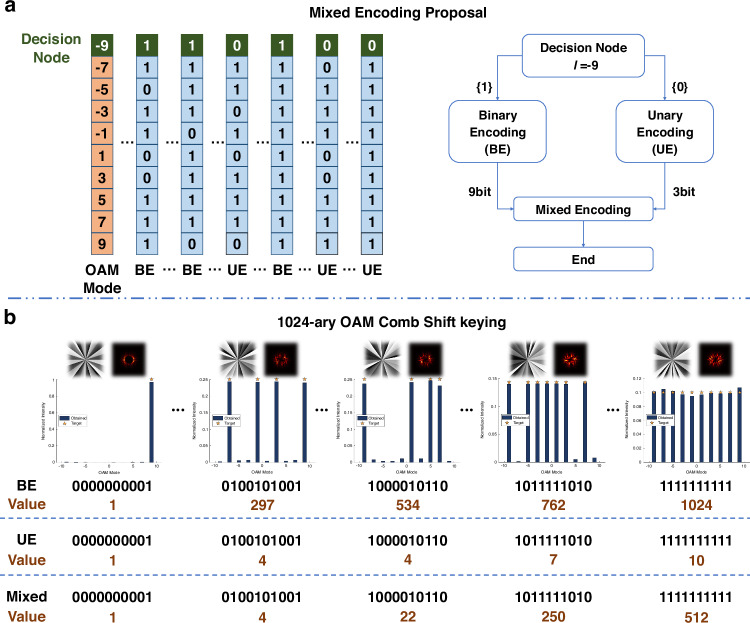
Mixed encoding proposal in OAM shift keying. **a** Workflow of the proposed mixed encoding proposal, where the OAM mode with *l* = −9 serves as a decision node to dynamically select binary encoding (BE) and unary encoding (UE). **b** Comparison of 1024-ary OAM comb shift keying under binary encoding, unary encoding, and the proposed mixed encoding proposal

To enhance both security and robustness in high-dimensional OAM comb one-to-many multicasting, we propose a mixed encoding proposal that integrates unary and binary encoding within a unified OAM comb framework, as shown in Fig. 2a. The approach hierarchically maps the OAM comb space, where robustness-critical symbols are encoded in a low-density unary subspace, while capacity-demanding symbols are mapped to a dense binary subspace. The core idea is to employ the OAM mode with topological charge *l* = -9 as a discriminant bit that dynamically selects the encoding branch. Based on the state of this decision node, two distinct encoding mechanisms are achieved:

Branch I – Unary 3-bit encoding (discriminant = 0): When the *l* = −9 mode is inactive (0), the symbol is encoded using a count-based unary scheme. After excluding the discriminant mode, the remaining nine OAM modes (−7, −5, −3, −1, +1, +3, +5, +7, +9), form the encoding pool. In this branch, the encoded symbol is determined solely by the number of activated OAM modes, rather than by which specific modes are active. During encoding, at most eight OAM modes are activated simultaneously, yielding 2^3^ = 8 valid unary symbols corresponding to 3-bit information. The specific subset of activated OAM modes is randomly selected from the nine available modes, and no fixed correspondence exists between a particular OAM mode and a symbol value. As a result, different OAM activation patterns may represent the same symbol, provided that the total count of active modes is identical. This design ensures that only the cardinality of the active OAM set carries information, while the exact activation pattern remains intentionally randomized.

This count-based unary encoding introduces an intrinsic security feature: an unintended receiver may observe the presence of multiple OAM components but cannot infer the transmitted symbol without knowledge of the counting rule. In this sense, the information is count-only observable but pattern-obscured, significantly increasing resistance against unauthorized decoding based on conventional OAM-SK detection strategies. To further enhance decoding robustness, one OAM mode may be reserved as a guard state for error detection; symbols violating the maximum-activation constraint are treated as invalid.

Branch II—Binary 9-bit encoding (discriminant = 1): When the *l* = −9 mode is active (1), the system switches to a binary encoding scheme across the remaining nine OAM modes. Each mode represents one bit, if the OAM mode is present, it denotes 1, otherwise, it denotes 0, yielding a total encoding capacity of 2^9^ = 512 symbols (9-bit). This enables dense, high-dimensional representation of information with a single holographic modulation.

Although the *l* = −9 OAM mode is used as the discriminant bit in our demonstration, the proposed mixed encoding strategy is fully general and can be implemented with any designated OAM mode within the comb. By introducing this mixed encoding proposal, the original 1024-ary OAM shift-keying scheme is extended to simultaneously exploit the robustness of unary encoding^29^ (low complexity and high error tolerance) and the high capacity of binary encoding, as shown in Fig. 2b. From a security perspective, the discriminant-bit mechanism effectively enlarges the decoding search space, since a receiver without prior knowledge of the discriminant mode must test multiple decoding branches, thereby increasing the complexity of unauthorized decoding. The introduction of a single discriminant bit reduces the encoding dimensionality by only 10% while providing an additional verification constraint for decoding. By dynamically selecting the encoding branch through the discriminant mode, the proposed approach enables flexible trade-offs among encoding dimensionality and transmission security, making it particularly well-suited for high-dimensional OAM comb multicasting systems.

Within this framework, the discriminant bit functions primarily as a verification indicator rather than the primary information carrier. Therefore, occasional misidentification of the discriminant mode caused by noise or modal crosstalk does not necessarily lead to catastrophic symbol failure. The encoded information is fundamentally contained in the overall OAM comb modal spectrum, which can still be partially recovered even if the discriminant bit is incorrectly detected. Instead, the introduction of the discriminant mechanism mainly provides an additional verification layer that increases the decoding complexity for unintended receivers while maintaining robust decoding performance for authorized users.

### Single-shot phase-only multiple OAM comb holography

From the perspective of one-to-many optical networking, the dimensionality of OAM-based data-transmission systems can be expanded through two complementary routes: (i) increasing the number of independently addressable spatial channels to support multiple users, and (ii) increasing the encoding dimensionality within each channel by using structured OAM combs. In this work, we focus on OAM comb as the high-dimensional symbols and exploit both routes simultaneously to construct a one-to-many multicasting framework. Specifically, multiple OAM modes are superposed to form structured OAM combs, while parallel multicasting channels are generated through spatial diffraction orders.

The key physical requirement for one-to-many OAM comb multicasting is the ability to simultaneously generate multiple high-dimensional OAM combs within a compact and reconfigurable optical architecture. However, existing OAM modulation approaches typically rely on cascaded optical elements or multi-path interferometric configurations, which impose severe limitations on system scalability and reconfigurability. To overcome these constraints, we develop a single-shot phase-only holographic modulation framework driven by a hybrid intelligent strategy. Unlike conventional OAM modulation schemes that rely on cascaded optical elements or multi-path interferometric architectures, the proposed approach enables direct synthesis of complex multi-mode OAM combs using a single phase-only hologram, thereby avoiding bulky optical setups and significantly reducing system complexity. Specifically, a multi-scale U-Net (MSUNet)^30^ is employed to learn the complex mapping between target OAM comb distributions and phase-only holograms, enabling efficient generation of high-dimensional OAM combs. In parallel, scalar diffraction theory is incorporated to accurately regulate the energy distribution and spatial separation among different diffraction orders. This hybrid framework leverages the pattern-learning capability of deep neural networks and the physical fidelity of diffraction modeling, enabling collaborative manipulation of OAM modes, relative modal weights, and diffraction orders within a single phase-only hologram.

**Fig. 3:**
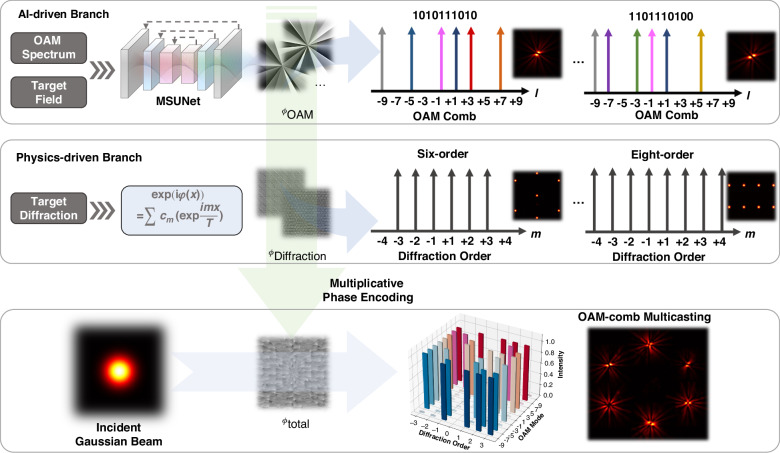
Hybrid intelligent strategy enabled a single-shot phase-only multiple OAM comb hologram. An AI-driven OAM comb phase design branch and a physics-guided diffraction-order optimization branch are coherently fused to enable simultaneous multicasting of OAM comb within a single phase-only hologram

Figure 3 illustrates the overall framework of the proposed hybrid intelligent strategy for single-shot phase-only multiple OAM comb holography. The framework is composed of two complementary branches, each targeting a distinct degree-of-freedom essential for scalable one-to-many OAM comb multicasting. In the AI-driven branch, the MSUNet learns the non-linear mapping between target OAM comb complex field and its corresponding phase-only holograms (${\phi }_{{OAM}}$). The objective function^31^ is defined to minimize the root-mean-square error (RMSE) between the reconstructed and target OAM comb spectrum, enabling efficient tailoring of structured OAM combs. In the physics-driven branch, diffraction-order modulation based on scalar diffraction theory enables the generation of parallel spatial channels required for one-to-many multicasting (${\phi }_{{Diffraction}}$). The objective function is constrained by the field-matching discrepancy. The two-phase components are subsequently fused through multiplicative phase encoding to form a single phase-only hologram, which simultaneously tailor the OAM comb structure and the spatial diffraction order (see Supplementary Note 1 for more details).

After the network training stage, phase-only hologram generation requires only a single forward inference of the trained model. In our implementation, the average inference time for generating a hologram is approximately 30 ms on a standard GPU platform (see more implementation details in Supplementary Note 2), while the physics-guided diffraction-order modulation step only involves analytical phase calculations and introduces negligible additional computational overhead. This generation time is comparable to the refresh rate of typical spatial light modulators (60–120 Hz), indicating that the proposed framework is compatible with near real-time hologram updating in practical optical communication systems.

This hybrid strategy effectively decouples the manipulation of the modal and spatial degrees of freedom while preserving physical consistency, enabling compact, reconfigurable, and scalable generation of multiple high-dimensional OAM combs in a single-shot holographic modulation process. By separating modal synthesis from spatial routing, the proposed framework allows each component to be independently optimized, achieving a favorable balance between generation speed and reconstruction fidelity. As a result, the hybrid strategy outperforms conventional AI-only or physics-only holographic design approaches.

### Experiment demonstration

**Fig. 4:**
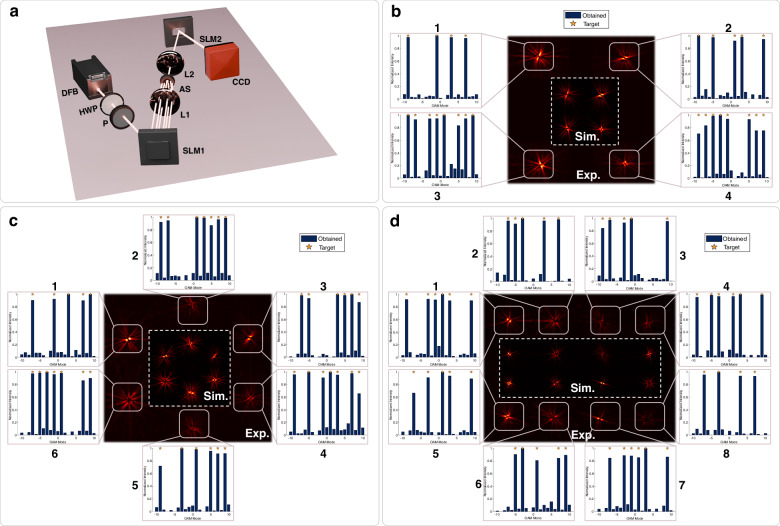
Experimental results of OAM comb multicasting. **a** The experimental setup. DFB, a 1617 nm distributed feedback laser diode, HWP half-wave plate, P polarizer, SLM1, SLM2 liquid-crystal spatial light modulator, L1, L2 plano-convex lenses with focal length 200 mm, AS aperture stop; CCD infrared CCD camera. Experimental visualizations and corresponding measured OAM spectrum for (**b**) one-to-four, (**c**) one-to-six, and (**d**) one-to-eight multicasting configurations, demonstrating scalable and high-fidelity OAM comb tailoring across multiple diffraction orders

The experiment setup is illustrated in Fig. 4a. A programmable phase-only modulation system based on two spatial light modulators (SLM) is constructed to experimentally validate the proposed high-dimensional OAM-comb multicasting framework. The generated OAM-comb carriers are encoded by the first SLM and relayed through a 4-*f* optical system. By adjusting the aperture stop (AS) located at the Fourier plane, the system can switch between OAM-comb tailoring and encrypted multicasting. For OAM comb tailoring performance evaluation, the AS is fully opened, allowing all diffraction orders of the generated OAM comb to pass through. In this configuration, SLM2 is used for OAM spectrum measurement by sequentially loading a set of conjugate spiral phases^32^ (see Supplementary Note 3 for more details). For the encrypted multicasting performance evaluation, the AS is partially closed. By adjusting the blazed grating parameters on SLM1, each multicast channel is sequentially directed through the aperture stop. In this mode, SLM2 loads a series of Dammann gratings to individually decode each diffraction order (see Supplementary Note 4 for more details).

### Tailoring performance

We first evaluate the capability of tailoring high-dimensional OAM comb multicasting. Multicasting transmission is experimentally demonstrated across 4, 6, and 8 channels, each supporting 1024-dimensional OAM-shift-keyed symbols, as shown in Fig. 4b-d.

By encoding various conjugate spiral phases on SLM2, following OAM back conversion theory^33,34^, the OAM modes can be analyzed. The measured OAM spectra exhibit excellent agreement with simulations, confirming accurate reconstruction of the targeted multi-mode structures. The average RMSE between the simulated and experimentally retrieved OAM spectra is 0.069 (Table 1), indicating high precision modulation across all channels. To further quantify the similarity between the generated and target OAM combs, we additionally introduce a fidelity metric^30^, inspired by the density-matrix formalism widely used for evaluating multi-mode states. The fidelity measures the overlap between the experimentally obtained and target OAM spectral distributions, providing a global indicator of modal agreement. As summarized in Tab. 1, the measured OAM combs exhibit high fidelity (about 0.9) across different diffraction orders (see Supplementary Note 5 for more details), further confirming the accurate tailoring performance of the proposed scheme. The fidelity remains consistently high even as the comb dimensionality increases, indicating that the modal quality of individual OAM components is well preserved during the OAM comb tailoring process.

**Table 1:** Measured RMSE, Fidelity and efficiency of OAM comb for each diffraction order

	4-order		6-order		8-order	
	RMSE	Fidelity	RMSE	Fidelity	RMSE	Fidelity
1	0.041	0.855	0.052	0.867	0.068	0.886
2	0.060	0.856	0.073	0.871	0.053	0.901
3	0.087	0.891	0.049	0.920	0.060	0.872
4	0.115	0.923	0.106	0.855	0.049	0.905
5	/	/	0.080	0.892	0.089	0.873
6	/	/	0.057	0.918	0.084	0.841
7	/	/	/	/	0.078	0.893
8	/	/	/	/	0.048	0.867
Efficiency	68.7%		69.0%		60.7%	

We additionally characterize the diffraction efficiency of the generated OAM combs for 4-, 6-, and 8-channel multicasting configurations. The average diffraction efficiency, defined as the ratio of the total optical power carried by all target OAM comb diffraction orders to the incident optical power illuminating the modulator, reaches 68.7%, 69.0%, and 60.7% for the 4-, 6-, and 8-order multicasting configurations, respectively. The remaining efficiency limitation mainly originates from practical diffraction factors, including finite SLM pixel resolution, and unavoidable power leakage into undesired diffraction orders. Such scalability in efficiency is essential for extending the proposed framework toward larger-scale multicasting systems. These results verify that the proposed hybrid holographic framework can reliably generate high-dimensional OAM comb signals across multiple spatial channels, providing a scalable physical platform for high-capacity optical multicasting and structured-light-based data transmission.

Despite the demonstrated scalability of the proposed multicasting framework, several practical factors may ultimately constrain the achievable system size. First, the number of multicast channels is partially limited by the spatial resolution of the SLM, since higher multicast orders require holograms with increasingly high spatial-frequency components. Second, maintaining sufficient angular separation between diffraction orders becomes more challenging as the channel number increases, potentially leading to spatial overlap or crosstalk in the Fourier plane. Third, the field of view of the detection system may restrict the number of spatially separated channels that can be simultaneously captured by CCD camera. These practical considerations define the current system limits, while further improvements in high-resolution SLM and array detectors are expected to extend the achievable multicasting scale.

### Encrypted multicasting performance

**Fig. 5:**
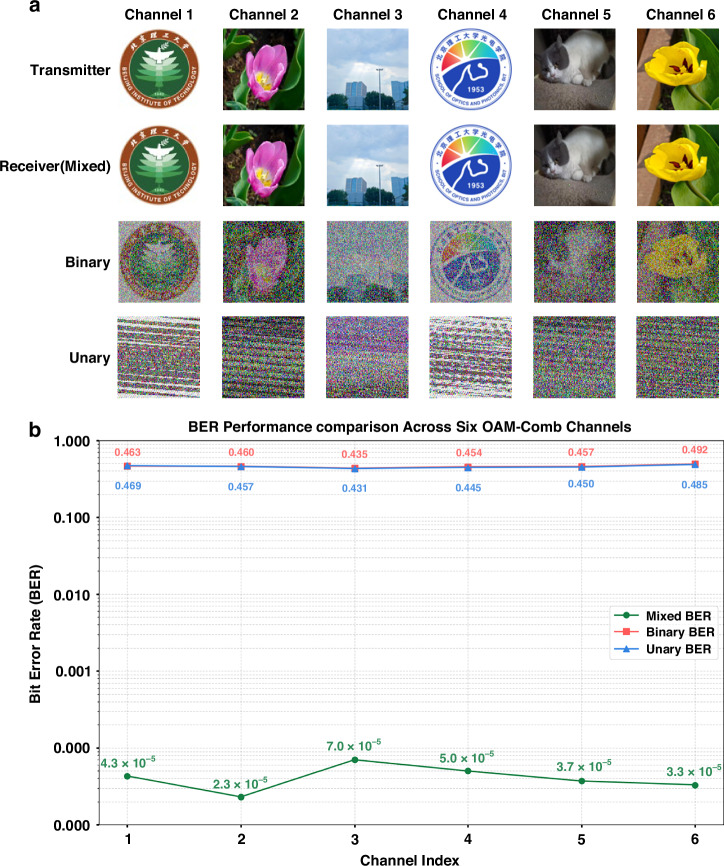
Multicasting results and BER performance analysis. **a** One-to-six OAM comb multicasting transmission results under mixed, binary, and unary decoding. **b** BER performance across the six OAM comb channels of mixed, binary, and unary decoding

Combined with the mixed encoding proposal described above, this capability enables simultaneous high-dimensional data multicasting across multiple spatial channels. To evaluate the encrypted communication capability of the proposed hybrid intelligent OAM comb multicasting system, we conduct a one-to-six parallel data transmission experiment, representing a typical multi-user optical communication scenario. In this experiment, six independent image contents—including institutional logos, floral patterns, natural scenes, and animal photographs—are simultaneously mapped onto six OAM comb multicast channels using the proposed mixed encoding proposal. All channels are jointly processed through the single-shot holographic modulation driven by the hybrid intelligent strategy, resulting in a single phase-only hologram for transmission. At the receiver, a DVG-based decoding module spatially separates the six OAM comb channels into distinct diffraction orders, allowing independent detection and decoding without introducing additional optical paths or active switching elements. The second row of Fig. 5a presents the reconstructed images obtained under the mixed encoding strategy. All channels exhibit high visual fidelity, with well-preserved structural details and texture information, and no observable inter-channel crosstalk. These results confirm that the proposed OAM comb multicasting framework maintains effective channel isolation even under simultaneous transmission of high-dimensional symbols.

To further assess the security of the mixed encoding strategy, we compare the decoding performance with pure binary and pure unary encoding schemes under the same experimental conditions. Notably, we assume an adversarial scenario in which an eavesdropper correctly guesses the encoding bit length (10 bits per symbol). Even under this favorable assumption, reliable image reconstruction cannot be achieved using either binary or unary encoding alone, as shown in the last two rows of Fig. 5a. In contrast, the mixed encoding strategy successfully combines the robustness of unary encoding with the high capacity of binary encoding through a hierarchical OAM comb mapping. By employing a designated OAM mode as a discriminant bit to dynamically select the decoding branch, the proposed scheme enables adaptive utilization of the available OAM dimensionality, which is particularly advantageous in multicasting scenarios involving simultaneous multi-channel transmission.

Quantitatively, the BER performance of the six multicast channels under different encoding schemes is summarized in Fig. 5b. Under the proposed mixed encoding proposal, all channels achieve BERs on the order of 10⁻⁵, confirming reliable and stable high-dimensional symbol decoding. In contrast, both binary and unary encoding schemes yield decoding accuracies close to random guessing (approximately 50%), even when the encoding dimensionality is correctly assumed. Furthermore, the consistent BER performance across all multicast channels demonstrates uniform energy distribution and effective mode separation enabled by the designed holograms and the DVG demultiplexing module. It is important to emphasize that the proposed hybrid intelligent framework is independent of the specific OAM indices. Different OAM mode distributions can represent the same encoded symbol, demonstrating the flexibility of the OAM comb-based transmission protocol. The choice of OAM comb $l\,=\,\{-9,-7,-5,-3,-1,+1,+3,+5,+7,+9\}$ in this work serves as a representative example. The system remains equally effective when utilizing other settings. The discriminant mode serves as a logical switch rather than a physical constraint, allowing the protocol to be adaptively reconfigured for different OAM comb configurations depending on the specific requirements of the secure communication link.

Overall, these results demonstrate that the proposed hybrid-intelligent OAM comb multicasting system simultaneously achieves high-dimensional encoding, low inter-channel crosstalk, and reliable decoding performance within a compact optical architecture. The demonstrated mixed encoding strategy and single-shot holographic modulation provide a practical and scalable solution for one-to-multiple optical communications, with strong potential for future high-capacity spatial-mode networks.

## Discussion

In this work, we introduce a hybrid intelligent strategy for high-dimensional OAM comb multicasting and demonstrate its capability through both multi-channel modulation and real-time communication experiments. By integrating deep learning with an iterative algorithm, we design phase-only holograms that directly generate multiple structured OAM combs within a single-shot modulation process. This approach overcomes the long-standing limitations of bulky multi-path OAM systems and enables compact, scalable, and high-fidelity high-dimensional encoding. We experimentally validate multicasting across 4, 6, and 8 parallel channels, each supporting a 1024-dimensional OAM encoding space, demonstrating excellent agreement with simulations, and confirming the robustness of the proposed modulation scheme. Building upon this high-dimensional capability, we further implement a mixed encoding strategy that enhances the security of one-to-multiple OAM comb multicasting, enabling safe and stable high-dimensional transmission in multi-user scenarios. A 6-channel OAM comb communication prototype successfully delivered distinct image streams in real time with a BER below 7 × 10⁻⁵, verifying both accuracy and stability of the decoding process. In addition, neither single-state nor multi-state decoding can reliably reconstruct the transmitted information, indicating inherent resistance to unauthorized decoding and enhanced transmission security. The demonstrated compatibility of the proposed approach highlights the strong potential of OAM comb multicasting as a new degree-of-freedom for scalable and reliable one-to-many optical communication networks. Overall, this hybrid intelligent OAM comb framework provides an effective route toward compact, high-dimensional, and multi-user optical communication architectures, opening opportunities for next-generation large-scale optical communication networks and advanced OAM-based optical information processing.

## Materials and methods

### Training configuration for OAM comb holography

The training dataset generation follows the procedure described in MSUNet^30^. The dataset consists of simulated target OAM comb field distributions, including both intensity and phase patterns. The OAM spectrum is defined within the range$\,l\in [-\mathrm{10,10}]\text{}$ where different OAM comb configurations are generated by randomly selecting multiple OAM modes within this range, such as *l*$=\,\left\{-9,-7,\ldots ,\mathrm{7,9}\right\}$. The modal amplitudes are randomly assigned while maintaining normalized energy distribution. For each OAM spectrum, the corresponding complex field is calculated, and the intensity and phase distributions are extracted as the network inputs. The spatial resolution is configured at 1080 × 1080 pixels with an active area of 10 mm × 10 mm. A total of 11,469 data pairs are generated. The dataset is divided into training and testing sets using an 8:2 split. For network initialization, ImageNet-pretrained weights are employed for the backbone layers to accelerate convergence. The remaining convolution layers are initialized using a standard normal distribution with a standard deviation of 0.01, while other parameters are initialized using the Xavier initialization scheme. During training, a batch size of 8 is used, and the model is trained on four NVIDIA GTX 6000 GPUs. The optimization is performed using stochastic gradient descent (SGD) with an initial learning rate of 1 × 10^−4^, a momentum of 0.9, and a weight decay of 5 × 10^−4^. All images are preprocessed and resized to a uniform resolution before training to ensure data consistency. In addition, a learning-rate decay strategy is applied, where the learning rate is adaptively adjusted according to the validation performance, further improving convergence speed and training stability. After training, phase-only hologram generation requires only a single forward pass of the neural network. On a standard GPU platform, the average inference time per hologram is approximately 30 ms, which is comparable to the refresh rate of typical spatial light modulators. This enables near real-time hologram updating in practical systems.

### Details of the experimental setup

The experimental setup is shown in Fig. 4a. A 1617 nm distributed feedback (DFB) laser diode is employed as the source to generate Gaussian beams. The beam is converted to linear polarization by passing sequentially through a half-wave plate (HWP) and a polarizer (P). The polarized beam is then incident on the first spatial light modulator (SLM1, Holoeye PLUTO-TELCO-013-C), which displays the phase-only holograms for generating the OAM comb multicasting carriers. The modulated beam from SLM1 is relayed to a second spatial light modulator (SLM2, Holoeye PLUTO-TELCO-013-C) via a 4-f imaging system composed of two lenses with focal lengths of 200 mm. An aperture stop (AS) is placed at the Fourier plane of the 4-f system and serves to switch between encoding and decoding operational modes. Finally, the far-field intensity distributions of all the beams are recorded using an infrared CCD camera (Cobra2000-CL1240-130VT-00, LUSTER).

## Supplementary information

Supplementary materials for Hybrid intelligent strategy driving high-dimensional encrypted orbital angular momentum comb multicasting towards optical data-transmission networks

## Data Availability

The data that support the findings of this study are available within the paper and the supplementary. Additional data related to this paper are available from the corresponding authors upon reasonable request.
