# Supplementary material for: Hybrid intelligent strategy driving high-dimensional encrypted orbital angular momentum comb multicasting towards optical data-transmission networks
Source: Light Sci Appl. 2026 Aug 5;15:339. doi: 10.1038/s41377-026-02386-3 (PMC13443203; doi:10.1038/s41377-026-02386-3)
Supplement: Supplementary file 1 [file 41377_2026_2386_MOESM1_ESM.docx]

**Supplementary Information for**

**Hybrid intelligent strategy driving high-dimensional encrypted orbital angular momentum comb multicasting towards optical data-transmission networks**

Shiyun Zhou^1,2,3^, Lang Li^1,2,3^, Jinyu Yang^1,2,3^, Shurui Zhang^1,2,3^, Zhiyuan Zhou^4,5^,

Chunqing Gao^1,3^ and Shiyao Fu^1,2,3,6, *^

*^1^ School of Optics and Photonics, Beijing Institute of Technology, Beijing 100081, China*

*^2^ Key Laboratory of Photoelectronic Imaging Technology and System, Ministry of Education of the People’s Republic of China, Beijing 100081, China*

*^3^ Key Laboratory of Information Photonics Technology, Ministry of Industry and Information Technology of the People’s Republic of China, Beijing 100081, China*

*^4^ Key Laboratory of Quantum Information, University of Science and Technology of China, Hefei, Anhui 230026, China*

*^5^ Synergetic Innovation Center of Quantum Information & Quantum Physics, University of Science and Technology of China, Hefei, Anhui 230026, China*

*^6^ National Key Laboratory on Near-surface Detection, Beijing, 100072, China*

** fushiyao@bit.edu.cn*

**Supplementary Note 1: Details of the single-shot phase-only OAM comb holography**

The proposed hybrid intelligent strategy combines an AI-driven hologram generation module with a physics-driven diffraction-order engineering module to realize single-shot generation of multiple OAM comb channels. The two components play complementary roles in the design pipeline. The AI-driven module learns the nonlinear mapping between a target OAM comb field and its corresponding phase-only hologram, enabling accurate reconstruction of high-dimensional OAM comb. The physics-driven module subsequently introduces diffraction manipulation by superimposing phase gratings that spatially separate multiple OAM comb channels into distinct diffraction orders. Unlike conventional iterative hologram optimization approaches, the two components are organized in a sequential pipeline rather than a joint optimization loop, allowing the hologram to be generated efficiently in a single forward inference step after network training.

Specifically, the AI-driven branch is constructed based on the MSUNet^30^, the input target OAM OAM comb field can be expressed as:

$$\begin{aligned} \text{Ψ}\left( \text{x} \right)\text{=}\sum_{\text{l}}^{\text{n}} \text{a}_{\text{l}}\text{LG}_{\text{l}}\left( \text{x} \right)\text{ }\#\left( \text{S1} \right) \end{aligned}$$

where $\text{LG}_{\text{l}}\left( \text{x} \right)$ denotes the Laguerre–Gaussian mode with topological charge *l*, and $\text{a}_{\text{l}}$corresponds to the amplitude of OAM mode $\left| \left. \text{l} \right\rangle\right.$, thus $\text{\{}\left| \text{a}_{\text{l}} \right|^{\text{2}}\text{\}}$, forming the OAM spectrum. The network adopts a dual-branch design, consisting of an intensity branch and a phase branch, which independently extract feature representations from the target intensity and phase patterns, respectively. Each branch employs the U-shape neural network $\text{f}_{\text{unet}}\left( \text{·} \right)$ as a feature extractor. The extracted intensity and phase features are subsequently fed into a multi-scale fusion learning module. This module employs a series of up-sampling operations to capture features at different spatial resolutions, followed by down-sampling layers to realign the spatial dimensions. The generate phase-only hologram $\phi_{OAM}$ reads:

$$\begin{aligned} \text{ϕ}_{\text{OAM}}\text{=}\text{α}\sum_{\text{i}\text{=1}}^{\text{m}} \text{p}_{\text{i}}\text{·}\text{f}_{\text{unet}}\left( \text{I} \right)\text{+}\text{β}\sum_{\text{i}\text{=1}}^{\text{m}} \text{q}_{\text{i}}\text{·}\text{f}_{\text{unet}}\left( \text{P} \right)\text{ }\#\left( \text{S2} \right) \end{aligned}$$

with $\text{α}$ and $\text{β}$ the hyper-parameters initially set as $\text{α}\text{=0.2}$ and $\text{β}\text{=0.8}$, respectively. $\text{p}$and $\text{q}$ denote scale factors. *m* is the number of scaling layers. $\text{I}$ and $\text{P}$ denote the input intensity and phase pattern data, respectively. Since no ground-truth holograms exist, we employ a physics-informed loss. The generated $\text{ϕ}_{\text{OAM}}$ is passed through an angular spectrum transmission layer, and the resulting field is analyzed by a pre-trained DRN^31^ to predict the output OAM spectrum.

$$\begin{aligned} \left\{ \left| \text{a}_{\text{l}} \right|^{\text{2}} \right\}\text{=}\text{f}_{\text{DRN}}\left( \text{f}_{\text{AS}}\left( \text{ϕ}_{\text{OAM}} \right) \right) \#\left( \text{S3} \right) \end{aligned}$$

here, the function $\text{f}_{\text{DRN}}\text{(·)}$ refers to the process of the pre-trained DRN model and $\text{f}_{\text{AS}}\text{(·)}$ represents the angular spectrum transmission. The difference between this output and the target OAM comb distribution is used as a constraint for loss backward, addressing the lack of ground-truth in supervised training and enhancing the network interpretability, achieving a reliable network training. The root mean square error (RMSE) is employed as loss function to evaluate the difference:

$$\text{Loss=}\text{L}^{\text{RMSE}}\left( \left\{ \left| \text{a}_{\text{l}} \right|^{\text{2}} \right\}\text{,}\hat{\left\{ \left| \text{a}_{\text{l}} \right|^{\text{2}} \right\}} \right)\text{=}\sqrt{\frac{\text{1}}{\text{2n+1}}\sum_{\text{l}\text{=-n}}^{\text{+n}} \left( \left\{ \left| \text{a}_{\text{l}} \right|^{\text{2}} \right\}\text{-}\hat{\left\{ \left| \text{a}_{\text{l}} \right|^{\text{2}} \right\}} \right)^{\text{2}}} \text{ }\left( \text{S4} \right)$$

with n the OAM mode range.

The physics-driven branch is based on scalar diffraction theory and is used to design phase-only holographic gratings for tailoring diffraction orders in OAM comb multicasting. In a one-dimensional (1D) holographic grating design, the objective is to determine a continuous phase-only function $\text{φ}\left( \text{x} \right)$ that generates a predefined set of diffraction orders. The phase grating can be expressed through a Fourier expansion as:

$$\begin{aligned} \exp\left( \text{i}\text{ϕ}_{\text{Diffraction}}\text{(}\text{x}\text{)} \right)\text{=}\sum\text{c}_{\text{m}}\exp\left( \frac{\text{imx}}{\text{T}} \right)\#\left( \text{S5} \right) \end{aligned}$$

where m is the diffraction order and T is the grating constant. The Fourier coefficients *c_m_* are given by

$$\begin{aligned} \text{c}_{\text{m}}\text{=}\frac{\text{T}}{\text{2}\text{π}}\int\exp\left( \text{i}\text{ϕ}_{\text{Diffraction}}\text{(}\text{x}\text{)} \right)\text{∙}\exp\left( \frac{\text{imx}}{\text{T}} \right)\text{d}\text{x}\#\left( \text{S6} \right) \end{aligned}$$

The above equations indicate that, by specifying the target diffraction orders *m*, the corresponding phase distribution $\text{ϕ}_{\text{Diffraction}}\text{(}\text{x}\text{)}$ can be uniquely determined. Regarding the target energy distribution among diffraction orders, this is enforced through the phase-grating coefficients, which control the relative diffraction efficiencies of the multiplexed channels. Since the grating parameters are analytically determined, the energy distribution can be directly adjusted by modifying the corresponding complex weighting coefficients. Specifically, we utilize a physics-constrained loss function based on the RMSE between the target and simulated diffraction fields. Since the grating parameters are determined via this physics-guided iterative process, the energy distribution can be deterministically tuned by adjusting the corresponding weighting coefficients to ensure high uniformity and efficiency across all multicast channels.

Finally, by employing a gradient descent algorithm, the final total hologram is obtained by multiplexing the phase components:

$$\begin{aligned} \text{ϕ}_{\text{total}}\text{=}\text{arg}\text{(}\sum\text{c}_{\text{m}}\exp\left( \text{i}\left( \text{ϕ}_{\text{OAM}}\text{+}\text{ϕ}_{\text{Diffraction}} \right) \right)\text{)}\#\left( \text{S7} \right) \end{aligned}$$

As an ablation, AI-only holograms fail to manipulate diffraction orders, and physics-only holograms lack precise high-dimensional OAM reconstruction. The hybrid approach simultaneously achieves both objectives, confirming the complementary advantages.

Fig.S1 presents the manipulation results of four-, six-, and eight-order OAM comb multicasting enabled by the proposed single-shot phase-only OAM comb holography. For each multicasting order, the tailoring OAM spectra show excellent agreement with the designed target distributions, confirming accurate manipulate over both the activated OAM modes and their relative amplitudes. The corresponding far-field intensity patterns further verify stable spatial separation among different diffraction orders, indicating reliable parallel channel generation.

These results demonstrate that the proposed holographic modulation scheme is capable of robustly encoding and decoding arbitrary high-dimensional OAM comb symbols within a single phase-only modulation step. By extending the binary combinations of the addressed OAM modes within the comb, the demonstrated four-, six-, and eight-order multicasting cases collectively validate the scalability of the method toward 1024-ary OAM shift keying. This capability establishes a solid foundation for high-capacity, high-dimensional OAM-based communication and multicasting systems.

**Fig. S1.** Single-shot phase-only OAM comb multicasting performance. Simulated manipulation results of four-, six-, and eight-order OAM comb multicasting under the proposed single-shot phase-only holographic modulation. Left panels show the far-field intensity distributions of the tailoring OAM comb beams for different multicasting orders. Right panels display the corresponding normalized OAM spectra, where the obtained modal amplitudes (bars) agree well with the target distributions (stars). The results confirm accurate OAM comb tailoring and support scalable high-dimensional OAM shift keying up to 1024 symbols.

**Supplementary Note 2: Implementation details**

The main optical parameters used in both simulation and experiment are summarized in Table S1, while the diffraction-order design parameters for the 4-, 6-, and 8-order configurations are provided in Tables S2–S4, respectively.

Tab.S1 Main optical parameters

| Parameter | Value |
| --- | --- |
| Laser wavelength | 1617 nm |
| Spatial grid size | 1080 |
| Simulation window | 10 mm |
| Gaussian beam waist radius | 1.5 mm |
| Initial propagation distance | 0 |
| Beam propagation distance | 100 mm |

Tab.S2 Four-order diffraction configuration

|  | T*_x_* (mm) | T*_y_* (mm) | Phase coefficient |
| --- | --- | --- | --- |
| 1 | 0.12 | 0.12 | 1 |
| 2 | 0.12 | -0.12 | i |
| 3 | -0.12 | 0.12 | -1 |
| 4 | -0.12 | -0.12 | -i |

Tab.S3 Six-order diffraction configuration

|  | T*_x_* (mm) | T*_y_* (mm) | Phase coefficient |
| --- | --- | --- | --- |
| 1 | 1.56 | 0.078 | 0.853 |
| 2 | 0.078 | 0.13 | 1.1i |
| 3 | -0.078 | 0.13 | -1.1 |
| 4 | 1.56 | -0.078 | 0.853i |
| 5 | -0.078 | -0.156 | 1.10 |
| 6 | 0.078 | -0.156 | -1.1i |

Tab.S4 Eight-order diffraction configuration

|  | T*_x_* (mm) | T*_y_* (mm) | Phase coefficient |
| --- | --- | --- | --- |
| 1 | -0.27 | 0.18 | 1.151 |
| 2 | -0.09 | 0.18 | 0.75i |
| 3 | 0.09 | 0.18 | -0.75 |
| 4 | 0.27 | 0.18 | -1.151i |
| 5 | -0.27 | -0.18 | 1.151 |
| 6 | -0.09 | -0.18 | 0.75i |
| 7 | 0.09 | -0.18 | -0.75 |
| 8 | 0.27 | -0.18 | -1.151i |

**Supplementary Note 3: Analysis of OAM combs**

The OAM comb spectrum measurement for determining the mode amplitude distribution is performed via an OAM back-conversion method. This process involves sequentially encoding a set of conjugate (anti-spiral) phases onto the tailoring OAM comb, followed by far-field intensity recording using a CCD camera. The underlying principle of back conversion can be described as follows: when a spiral phase with topological charge *-l* is applied, the corresponding OAM component with charge *l* in the OAM comb is converted to a Gaussian-like mode (*l*-*l*=0), producing a bright intensity spot at the beam center. In contrast, all other OAM components $l_{o}$ are transformed into residual vortex modes (*l*_0_-*l*≠0), which do not concentrate energy at the center. Consequently, by measuring the on-axis intensity of the back-converted beam for each applied spiral phase, the presence and relative amplitude of each OAM mode in the comb can be quantitatively determined. It should be noted that, although the target OAM comb employed in this work consists of ten modes {−9, −7, −5, −3, −1, +1, +3, +5, +7, +9}, the actual holographic modulation requires manipulation of a broader OAM-mode space spanning from −10 to 10, comprising a total of 21 modes. An experimentally captured back-converted intensity patterns for OAM comb measurement result is shown in Fig. S2. The order of the applied back-converting spiral phase is indicated in the upper-right corner of each inset. The gray dashed circle marks the sampling region used to extract intensity, which is treated as the measured amplitude of the corresponding OAM channel.

**Fig. S2.** Experimentally captured back-converted example patterns for OAM comb measurement. The topological charge of each back-converting spiral phase is labeled in the upper-right corner and the gray dashed circle indicates the sampling area.

**Supplementary Note 4: DVG-based decoding**

At the receiver side, a DVG–based decoding module is employed to demultiplex the simultaneously transmitted OAM comb multicast channels and recover the encoded high-dimensional symbols as shown in Fig.S3(a). The DVG introduces a designed phase profile that combines vortex phase modulation with Damman grating diffraction, enabling structured OAM modes to be spatially separated into distinct diffraction orders in the far field. After propagation, the spatially separated diffraction spots are recorded by an infrared CCD camera. For each diffraction order, the corresponding OAM spectrum is extracted by comparing with the standard encoding element patterns, as illustrated in Figs.S3(b-c), resulting in a reliable identification of the active OAM modes within the comb. Based on the recovered OAM spectrum, symbol decoding is performed according to the predefined mixed encoding proposal. Specifically, the designated discriminant OAM mode (*l*=-9) determines the decoding branch, while the remaining OAM comb components are interpreted using either unary or binary decoding.

Because the Damman grating provides near-uniform energy allocation across diffraction orders, the decoding performance remains stable as the number of multicast channels increases. Importantly, the DVG-based decoding scheme is inherently compatible with the proposed single-shot phase-only OAM comb holography. Both multicasting and demultiplexing are realized using programmable phase modulation without introducing additional bulk optical elements or interferometric paths. This fully phase-only and reconfigurable architecture enables scalable one-to-multiple communication and supports real-time switching between different multicast configurations.

**Fig. S3.** DVG-based decoding illustration. (a) Decoding result of a representative OAM comb symbol 0100001101 using the DVG-based scheme. The corresponding phase pattern encoded on the SLM is shown in the lower-left inset. The decoded OAM modes span topological charges from -10 to +10. (b) Far-field diffraction pattern corresponding to the standard code element, where the DVG spatially separates the OAM comb components into distinct diffraction orders. (c) Experimental decoding result after DVG-based demultiplexing under the antithetical code condition, demonstrating correct symbol discrimination and robustness of the decoding process.

**Supplementary Note 5: Experimental fidelity characterization**

To provide a more comprehensive and intuitive evaluation of the tailoring performance, we introduce an additional metric——fidelity $\text{F}$, inspired by the density-matrix formalism widely used in quantum optics to evaluate multi-mode states. In this framework, the generated OAM comb $\left| \left. \text{Ψ} \right\rangle\right.$ can be expressed as:

$$\begin{aligned} \left| \left. \text{Ψ} \right\rangle\right.\text{=}\sum_{\text{l}\text{=-n}}^{\text{+n}} \text{a}_{\text{l}}\left| \left. \text{l} \right\rangle\right. \#\left( \text{S8} \right) \end{aligned}$$

where $\left| \left. \text{l} \right\rangle\right.$ represents the OAM state and $\text{a}_{\text{l}}$ represents the corresponding normalized complex amplitude. Thus, the corresponding density matrix $\rho$ for the generated OAM comb is defined as:

$$\begin{aligned} \text{ρ}\text{=}\left| \left. \text{Ψ} \right\rangle\right.\left\langle\left. \text{Ψ} \right| \right. \#\left( \text{S9} \right) \end{aligned}$$

To quantify the similarity between the experimentally generated OAM comb and the target OAM comb, the state fidelity is calculated as:

$$\begin{aligned} \text{F}\text{=}\left\langle\left. \text{Ψ}_{\text{tar}} \right| \right.\text{ρ}_{\text{exp}}\left| \left. \text{Ψ}_{\text{tar}} \right\rangle\right.\text{ }\boldsymbol{\#}\left( \text{S10} \right) \end{aligned}$$

where $\left| \left. \text{Ψ}_{\text{tar}} \right\rangle\right.$ denotes the target OAM comb state and $\text{ρ}_{\text{exp}}$ denotes the density matrix reconstructed from the experimentally measured modal spectrum.

This fidelity metric quantitatively characterizes the similarity between the measured and target OAM modal distributions. A higher fidelity indicates a better agreement between the generated and desired OAM comb structures. The experimental modal intensity data used for fidelity evaluation are summarized in Tab. S5–S7, which list the target and measured OAM spectra for the 4-, 6-, and 8-order diffraction channels, respectively, covering the OAM mode range from $\text{l}\text{ }\text{=}\text{ }\text{-10}$ to $\text{10}$.

Tab. S5 Data results of OAM comb for 4 diffraction order.

|  | 1 | | 2 | | 3 | | 4 | |
| --- | --- | --- | --- | --- | --- | --- | --- | --- |
|  | Tar. | Exp. | Tar. | Exp. | Tar. | Exp. | Tar. | Exp. |
| -10 | 0 | 0.1260 | 0 | 0.0699 | 0 | 0.0817 | 0 | 0.0506 |
| -9 | 0.5000 | 0.4620 | 0.4472 | 0.4219 | 0.3536 | 0.3428 | 0.3536 | 0.3078 |
| -8 | 0 | 0.0807 | 0 | 0.0920 | 0 | 0.0454 | 0 | 0.0309 |
| -7 | 0 | 0.0704 | 0 | 0.1650 | 0.3536 | 0.3305 | 0.3536 | 0.3347 |
| -6 | 0 | 0.1219 | 0 | 0.0720 | 0 | 0.0534 | 0 | 0.0559 |
| -5 | 0 | 0.0393 | 0.4472 | 0.4163 | 0 | 0 | 0.3536 | 0.3637 |
| -4 | 0 | 0.0846 | 0 | 0.0372 | 0 | 0.1190 | 0 | 0.0957 |
| -3 | 0 | 0.1027 | 0 | 0.1023 | 0.3536 | 0.3329 | 0.3536 | 0.3664 |
| -2 | 0 | 0.0946 | 0 | 0.0867 | 0 | 0.0716 | 0 | 0.0935 |
| -1 | 0.5000 | 0.4676 | 0 | 0.1323 | 0.3536 | 0.3334 | 0.3536 | 0.3547 |
| 0 | 0 | 0.0543 | 0 | 0.0750 | 0 | 0.0652 | 0 | 0.1269 |
| 1 | 0 | 0 | 0.4472 | 0.4041 | 0.3536 | 0.3428 | 0 | 0 |
| 2 | 0 | 0.1140 | 0 | 0.0890 | 0 | 0.0616 | 0 | 0.0656 |
| 3 | 0.5000 | 0.4615 | 0.4472 | 0.4172 | 0 | 0.1614 | 0 | 0.1096 |
| 4 | 0 | 0.0758 | 0 | 0.1391 | 0 | 0.1331 | 0 | 0.0544 |
| 5 | 0 | 0.1225 | 0 | 0 | 0.3536 | 0.3132 | 0.3536 | 0.3539 |
| 6 | 0 | 0.0781 | 0 | 0.1106 | 0 | 0.1276 | 0 | 0.0623 |
| 7 | 0.5000 | 0.4583 | 0 | 0 | 0.3536 | 0.3331 | 0.3536 | 0.3182 |
| 8 | 0 | 0.0850 | 0 | 0.1029 | 0 | 0.0414 | 0 | 0.0863 |
| 9 | 0 | 0.0951 | 0.4472 | 0.4101 | 0.3536 | 0.3419 | 0.3536 | 0.3183 |
| 10 | 0 | 0.1244 | 0 | 0.0695 | 0 | 0.0840 | 0 | 0.0431 |
| ***F*** | 0.855 | | 0.856 | | 0.891 | | 0.923 | |

Tab. S6-1 Data results of OAM comb for 6 diffraction order.

|  | 1 | | 2 | | 3 | |
| --- | --- | --- | --- | --- | --- | --- |
|  | Tar. | Exp. | Tar. | Exp. | Tar. | Exp. |
| -10 | 0 | 0.1051 |  | 0.1210 | 0 | 0.0439 |
| -9 | 0 | 0.0834 | 0.3780 | 0.3469 | 0 | 0 |
| -8 | 0 | 0.0630 |  | 0.0732 | 0 | 0.0939 |
| -7 | 0.4472 | 0.4173 | 0.3780 | 0.3517 | 0.4082 | 0.3955 |
| -6 | 0 | 0.1097 | 0 | 0.0921 | 0 | 0.0969 |
| -5 | 0 | 0.1359 | 0 | 0.0978 | 0.4082 | 0.3863 |
| -4 | 0 | 0.0957 | 0 | 0.0896 | 0 | 0.0700 |
| -3 | 0 | 0 | 0 | 0 | 0 | 0 |
| -2 | 0 | 0.1152 | 0 | 0.1017 | 0 | 0.0394 |
| -1 | 0.4472 | 0.4215 | 0 | 0 | 0 | 0.0911 |
| 0 | 0 | 0.0975 | 0 | 0.1218 | 0 | 0.0714 |
| 1 | 0 | 0 | 0.3780 | 0.3610 | 0 | 0 |
| 2 | 0 | 0.1327 | 0 | 0.0947 | 0 | 0.0439 |
| 3 | 0.4472 | 0.4215 | 0.3780 | 0.3599 | 0.4082 | 0.3995 |
| 4 | 0 | 0.0574 | 0 | 0.1230 | 0 | 0.1087 |
| 5 | 0 | 0.0965 | 0.3780 | 0.3372 | 0.4082 | 0.3958 |
| 6 | 0 | 0.0413 | 0 | 0.0887 | 0 | 0.0732 |
| 7 | 0.4472 | 0.4132 | 0.3780 | 0.3545 | 0.4082 | 0.3995 |
| 8 | 0 | 0.1192 | 0 | 0.1222 | 0 | 0.1294 |
| 9 | 0.4472 | 0.4096 | 0.3780 | 0.3588 | 0.4082 | 0.3732 |
| 10 | 0 | 0.0382 | 0 | 0.0994 | 0 | 0.0583 |
| ***F*** | 0.867 | | 0.871 | | 0.920 | |

Tab. S6-2 Data results of OAM comb for 6 diffraction order.

|  | 4 | | 5 | | 6 | |
| --- | --- | --- | --- | --- | --- | --- |
|  | Tar. | Exp. | Tar. | Exp. | Tar. | Exp. |
| -10 | 0 | 0.0840 | 0 | 0.1050 | 0 | 0.0702 |
| -9 | 0.3780 | 0.3572 | 0.4082 | 0.3429 | 0 | 0 |
| -8 | 0 | 0.0581 | 0 | 0.0709 | 0 | 0.0776 |
| -7 | 0 | 0.0567 | 0 | 0 | 0.3780 | 0.3664 |
| -6 | 0 | 0.1062 | 0 | 0.0597 | 0 | 0.0467 |
| -5 | 0.3780 | 0.3648 | 0 | 0 | 0.3780 | 0.3684 |
| -4 | 0 | 0.0778 | 0 | 0.1009 | 0 | 0.1209 |
| -3 | 0 | 0 | 0.4082 | 0.4029 | 0.3780 | 0.3711 |
| -2 | 0 | 0.0972 | 0 | 0.0779 | 0 | 0.1039 |
| -1 | 0.3780 | 0.3483 | 0 | 0.1091 | 0.3780 | 0.3640 |
| 0 | 0 | 0.1272 | 0 | 0.1235 | 0 | 0.0914 |
| 1 | 0.3780 | 0.3643 | 0.4082 | 0.3994 | 0.3780 | 0.3676 |
| 2 | 0 | 0.1117 | 0 | 0.0691 | 0 | 0.0695 |
| 3 | 0.3780 | 0.3563 | 0 | 0 | 0 | 0 |
| 4 | 0 | 0.0994 | 0 | 0.1157 | 0 | 0.0869 |
| 5 | 0 | 0.1077 | 0.4082 | 0.3943 | 0 | 0 |
| 6 | 0 | 0.1562 | 0 | 0.0502 | 0 | 0.0941 |
| 7 | 0.3780 | 0.3608 | 0.4082 | 0.3867 | 0.3780 | 0.3453 |
| 8 | 0 | 0.1009 | 0 | 0.0611 | 0 | 0.0957 |
| 9 | 0.3780 | 0.2959 | 0.4082 | 0.3871 | 0.3780 | 0.3521 |
| 10 | 0 | 0.1245 | 0 | 0.1349 | 0 | 0.0643 |
| ***F*** | 0.855 | | 0.892 | | 0.918 | |

Tab. S7-1 Data results of OAM comb for 8 diffraction order.

|  | 1 | | 2 | | 3 | | 4 | |
| --- | --- | --- | --- | --- | --- | --- | --- | --- |
|  | Tar. | Exp. | Tar. | Exp. | Tar. | Exp. | Tar. | Exp. |
| -10 | 0 | 0.0714 | 0 | 0.1619 | 0 | 0.1293 | 0 | 0.0800 |
| -9 | 0.4082 | 0.3829 | 0 | 0 | 0.4472 | 0.3959 | 0.4082 | 0.3838 |
| -8 | 0 | 0.1126 | 0 | 0.1410 | 0 | 0.0773 | 0 | 0.0560 |
| -7 | 0 | 0 | 0.4472 | 0.4242 | 0.4472 | 0.4252 | 0 | 0 |
| -6 | 0 | 0.0786 | 0 | 0.0648 | 0 | 0.0916 | 0 | 0.1290 |
| -5 | 0 | 0.0983 | 0.4472 | 0.4136 | 0 | 0.0231 | 0.4082 | 0.3902 |
| -4 | 0 | 0.0905 | 0 | 0.0495 | 0 | 0.1029 | 0 | 0.0644 |
| -3 | 0.4082 | 0.3841 | 0.4472 | 0.4323 | 0.4472 | 0.4159 | 0.4082 | 0.3863 |
| -2 | 0 | 0.0649 | 0 | 0.0563 | 0 | 0.0556 | 0 | 0.1189 |
| -1 | 0.4082 | 0.3820 | 0 | 0 | 0.4472 | 0.4305 | 0 | 0 |
| 0 | 0 | 0.1685 | 0 | 0.0917 | 0 | 0.1319 | 0 | 0.1356 |
| 1 | 0.4082 | 0.3996 | 0 | 0 | 0 | 0.1460 | 0.4082 | 0.3863 |
| 2 | 0 | 0.0709 | 0 | 0.1477 | 0 | 0.0965 | 0 | 0.0962 |
| 3 | 0.4082 | 0.3788 | 0.4472 | 0.4243 | 0 | 0 | 0.4082 | 0.3929 |
| 4 | 0 | 0.0531 | 0 | 0.0273 | 0 | 0.0651 | 0 | 0.0763 |
| 5 | 0 | 0 | 0 | 0 | 0 | 0.0961 | 0 | 0 |
| 6 | 0 | 0.0780 | 0 | 0.0391 | 0 | 0.0941 | 0 | 0.0738 |
| 7 | 0 | 0.0949 | 0.4472 | 0.4284 | 0 | 0.0651 | 0 | 0 |
| 8 | 0 | 0.0761 | 0 | 0.0456 | 0 | 0.0811 | 0 | 0.0715 |
| 9 | 0.4082 | 0.3795 | 0 | 0 | 0.4472 | 0.4211 | 0.4082 | 0.3915 |
| 10 | 0 | 0.0985 | 0 | 0.0897 | 0 | 0.0194 | 0 | 0.0772 |
| ***F*** | 0.886 | | 0.901 | | 0.872 | | 0.905 | |

Tab. S7-2 Data results of OAM comb for 8 diffraction order.

|  | 5 | | 6 | | 7 | | 8 | |
| --- | --- | --- | --- | --- | --- | --- | --- | --- |
|  | Tar. | Exp. | Tar. | Exp. | Tar. | Exp. | Tar. | Exp. |
| -10 | 0 | 0.1024 | 0 | 0.0479 | 0 | 0.0814 | 0 | 0.0749 |
| -9 | 0 | 0 | 0 | 0 | 0 | 0 | 0 | 0.1490 |
| -8 | 0 | 0.1191 | 0 | 0.0623 | 0 | 0.0973 | 0 | 0.0719 |
| -7 | 0.4472 | 0.3642 | 0 | 0.1206 | 0.4082 | 0.3768 | 0.5000 | 0.4658 |
| -6 | 0 | 0.1234 | 0 | 0.0671 | 0 | 0.0785 | 0 | 0.1320 |
| -5 | 0 | 0.1024 | 0.4472 | 0.4141 | 0 | 0.1196 | 0 | 0 |
| -4 | 0 | 0.0867 | 0 | 0.0917 | 0 | 0.0998 | 0 | 0.1342 |
| -3 | 0.4472 | 0.4263 | 0.4472 | 0.4345 | 0.4082 | 0.3858 | 0.5000 | 0.4761 |
| -2 | 0 | 0.0931 | 0 | 0.1008 | 0 | 0.1381 | 0 | 0.1402 |
| -1 | 0 | 0 | 0 | 0 | 0.4082 | 0.3844 | 0 | 0 |
| 0 | 0 | 0.0911 | 0 | 0.1116 | 0 | 0.0739 | 0 | 0.1198 |
| 1 | 0.4472 | 0.4460 | 0.4472 | 0.3915 | 0.4082 | 0.3788 | 0 | 0 |
| 2 | 0 | 0.0490 | 0 | 0.0813 | 0 | 0.0481 | 0 | 0.0984 |
| 3 | 0.4472 | 0.4321 | 0 | 0.1405 | 0.4082 | 0.4087 | 0.5000 | 0.4610 |
| 4 | 0 | 0.0566 | 0 | 0.1054 | 0 | 0.0801 | 0 | 0.0705 |
| 5 | 0 | 0 | 0 | 0.1737 | 0 | 0 | 0 | 0 |
| 6 | 0 | 0.0924 | 0 | 0.0863 | 0 | 0.0859 | 0 | 0.0757 |
| 7 | 0 | 0.1215 | 0.4472 | 0.4000 | 0 | 0 | 0.5000 | 0.4599 |
| 8 | 0 | 0.0937 | 0 | 0.0753 | 0 | 0.1240 | 0 | 0.0599 |
| 9 | 0.4472 | 0.4218 | 0.4472 | 0.4114 | 0.4082 | 0.3809 | 0 | 0 |
| 10 | 0 | 0.0977 | 0 | 0.1421 | 0 | 0.0569 | 0 | 0.0775 |
| ***F*** | 0.873 | | 0.841 | | 0.893 | | 0.867 | |
